# Can Inhibin B Reflect Ovarian Reserve of Healthy Reproductive Age Women Effectively?

**DOI:** 10.3389/fendo.2021.626534

**Published:** 2021-04-14

**Authors:** Jingyi Wen, Kecheng Huang, Xiaofang Du, Hanwang Zhang, Ting Ding, Cuilian Zhang, Wenmin Ma, Ying Zhong, Wenyu Qu, Yi Liu, Zhiying Li, Song Deng, Aiyue Luo, Yan Jin, Jinjin Zhang, Shixuan Wang

**Affiliations:** ^1^Department of Obstetrics and Gynecology, Tongji Hospital, Tongji Medical College, Huazhong University of Science and Technology, Wuhan, China; ^2^Department of Obstetrics and Gynecology, Central Hospital of Wuhan, Tongji Medical College, Huazhong University of Science and Technology, Wuhan, China; ^3^Reproductive Medical Center, Henan Provincial People’s Hospital, Zhengzhou, China; ^4^Reproductive Medical Center, Foshan Maternal and Child Health Care Hospital, Foshan, China; ^5^Reproductive Medical Center, Chengdu Jinjiang Maternal and Child Health Hospital, Chengdu, China; ^6^Reproductive Medical Center, Shenyang Women’s and Children’s Hospital, Shenyang, China; ^7^Department of Obstetrics and Gynecology, Union Hospital, Tongji Medical College, Huazhong University of Science and Technology, Wuhan, China; ^8^Department of Obstetrics and Gynecology, Renhe Hospital, China Three Gorges University, Yichang, China; ^9^Department of Gynecology, Minda Hospital of Hubei Minzu University, Enshi, China

**Keywords:** Inhibin B, follicle-stimulating hormone, anti-Mullerian hormone, antral follicle count, ovarian reserve, fertility

## Abstract

**Objective:**

The reference range and potential value of inhibin B are still unclear and controversial. This study aimed to define the variation trend of inhibin B in healthy women with age and explore its value in the reflection of ovarian reserve.

**Methods:**

A total of 2524 healthy reproductive age women from eight medical institutes nationwide were recruited. The variation tendency of inhibin B with age was primarily established in the first group of 948 women and validated in another 605. We evaluated the relationship between inhibin B and classic ovarian reserve and function markers. The potency of inhibin B in predicting AFC <5-7 was also estimated and compared with FSH.

**Results:**

The nomogram showed that serum levels of inhibin B rapidly decreased after the age of 40. Inhibin B was positively correlated with AMH (R = 0.57, P < 0.001), AFC (R = 0.34, P < 0.001) and testosterone (R = 0.10, P = 0.002), and negatively correlated with FSH (R = -0.41, P < 0.001) and LH (R = -0.20, P < 0.001) and FSH/LH (R=-0.18, P < 0.001), while no correlation was found with PRL. Unexpectedly, Inhibin B (AUC = 0.74, P < 0.001 for the establishment population; AUC = 0.78, P < 0.001 for the validation population) had a slightly higher value than FSH (AUC = 0.71, P < 0.001 for the establishment population; AUC = 0.72, P < 0.001 for the validation population) in diagnosing AFC <5-7.

**Conclusions:**

For healthy reproductive age women, the decline of inhibin B can reflect decreased ovarian reserve effectively, having a good consistency with AMH and AFC. More importantly, inhibin B had an advantage in predicting AFC <5-7 compared with FSH, which suggested the potential of inhibin B in predicting ovarian response. These results will be helpful to the clinical application of inhibin B in the evaluation of female ovarian reserve and the assessment of their reproductive capacity. Trial registration: http://clinicaltrials.gov; NCT02294500.

## Introduction

Inhibin B, a heterodimeric glycoprotein that comprises an alpha subunit linked to a beta-B subunit, belongs to the superfamily of transforming growth factor-β. Secreted by granulosa cells of developing follicles, the non-steroidal hormone is well known for its property of suppressing follicle-stimulating hormone (FSH). A high level of inhibin B in the serum directly exerts negative feedback on the pituitary gland, leading to a decrease in FSH. Therefore, the higher level of serum inhibin B of reproductive age women is one of the important factors to maintain a low level of serum FSH. However, with the increase of their age, both the quality and quantity of ovarian follicles decrease, the level of serum inhibin B decreases gradually, and the inhibitory effect on FSH will be weakened, which is also one of the important reasons for the progressive increase of their serum FSH levels (1–3).

With continuous study, researchers gradually realized the importance of Inhibin B in female fertility. The findings of previous studies suggested that inhibin B may have certain clinical application potential in assessing the progress of ovarian aging, diagnosing premature ovarian failure (POF) or premature ovarian insufficiency (POI), evaluating the ovarian function of cancer survivors, and predicting assisted reproductive technology (ART) outcomes. Welt et al. found that the decrease in inhibin B was the earliest marker of the decline in follicle number across reproductive aging (4). Bidet et al. found that inhibin B was one of the predictive factors for the resumption of ovarian function in POF patients (5). Recently, Zhu and colleagues revealed that there was a significantly continuous decline in inhibin B accompanying the progress of POI (6). Studies on cancer survivors showed significantly lower inhibin B levels in cancer survivors (7, 8). However, other studies showed no significant difference between cancer survivors and controls (9, 10). Due to differences in the study populations, the inclusion criteria used, and the methodologies used in several laboratories, the conclusions of studies on inhibin B and ovarian response and ART outcomes varied (11–15). Collectively, studies on the clinical application value of inhibin B were still had inconsistent findings, and the evidence was insufficient.

Moreover, few studies have focused on the variation tendency and reference range of inhibin B in healthy reproductive age women, which is necessary to be determined, will contribute to a better assessment of ovarian function and facilitate the clinical application of inhibin B. Despite the potential value of inhibin B, the uniform normative data for female adults are rare worldwide. Besides, the relationship between inhibin B and other classical ovarian reserve markers including FSH, anti-Mullerian hormone (AMH), and antral follicle count (AFC) remains unclear to date. To define the variation trend of inhibin B in female adults with age, and explore its value in the reflection of ovarian reserve and function, we detected the levels of inhibin B in a group of reproductive age women and investigated whether a correlation exists between inhibin B and other important ovarian reserve and function makers including AFC, FSH, AMH, LH, prolactin (PRL), testosterone (T), and progesterone.

## Methods

### Study Centers

Since October 2011, a nationwide, standardized, systematic research protocol was used for women over 20 years old in this prospective and open-label study. A group of healthy Chinese females was recruited (n=2524) through advertisements to establish an ovarian reserve database that included clinical and biological factors. Six universities and eight medical institutes participated in this recruitment.

### Inclusion Criteria

This research included a questionnaire regarding fertility, family history, and climacteric complaints, as well as ultrasonography and blood examination. Volunteers were enrolled in the study if they met all of our criteria, which were also adopted in our previous studies (16, 17). The inclusion criteria were as follows: (1) for women <40 years old having regular menstrual cycles and for women >40 not required to have regular menstrual cycles considering that they may be in normal perimenopause or menopause; (2) no hormone therapy in the past 6 months; (3) no history of radiotherapy or chemotherapy; (4) no history of hysterectomy, oophorectomy, or any other type of ovarian surgery; (5) no ovarian cysts or ovarian tumors; (6) no known chronic, systemic, metabolic, or endocrine diseases such as hyperandrogenism or hyperprolactinemia.

### Ethics and Informed Consent

The clinical investigation followed the Declaration of Helsinki, and the protocol was approved by the Ethical Committee of Tongji Hospital, Tongji Medical College, Huazhong University of Science and Technology. All eligible patients gave written informed consent before entering this study. The clinical data from all patients came from the ovarian aging database v1.0 (http://clinicaltrials.gov; NCT02294500).

### Study Design

According to international standards, a reference range based on at least 120 individuals is the preferred method (18). After 54 women with missing values, 2 women older than 50 years, and 915 women who did not meet inclusion criteria strictly were excluded, the data from 1553 women were left for our study. The data from 948 women were employed to establish the reference range, and the remaining 605 were utilized for further validation.

**Figure 1:**
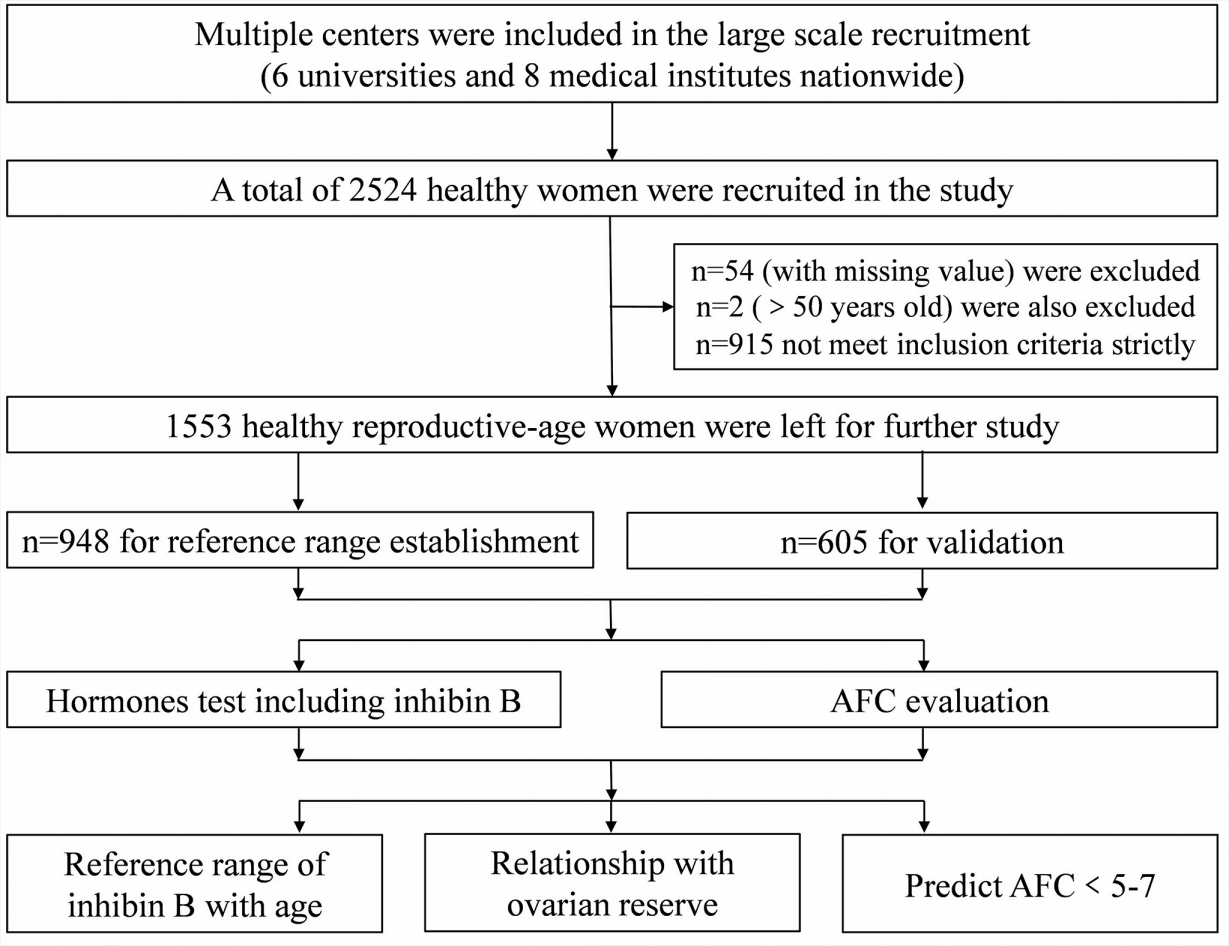
The flowchart of this study.

Each volunteer had a face-to-face interview using a prepared questionnaire. Blood samples from the follicular phase or day 2–5 of the menstrual cycle were drawn from an antecubital vein, clotted, and centrifuged, and the serum was aliquoted sterile and stored at −80°C until hormone analyses were performed. The flowchart of the study was shown in **Figure 1**.

### Hormones Assays

Serum levels of inhibin B, AMH, FSH, LH, estradiol (E2), PRL, progesterone, and T were evaluated. Levels of inhibin B and AMH were measured using inhibin B ELISA kits and AMH ELISA kits from Beckman Coulter Inc., which were described in the previous research (19). All serum measurements for the patients were performed in the same laboratory using the same assays. The assay was developed using a sequential application of sample, conjugated antibody, substrate, and amplifier, with the washing as instructed. Absorbance was read in a microplate reader at a wavelength of 450 nm, along with 620 nm used as a reference filter.

Sample concentrations of inhibin B were extrapolated from the standard curve using a cubic-cubic regression. The detection limit of the assay was 2.6 pg/mL, the intra- and inter-assay coefficients of variation were 3.8% and 5.2%, respectively. Samples with inhibin B concentrations less than the detection limit of the assay were assigned a value of 2.5 for analysis.

The levels of FSH, LH, E2, PRL, progesterone, and T were measured using a chemiluminescence-based immunometric assay on an ADVIA Centaur immunoassay system (Siemens Healthcare Diagnostics Inc., Tarrytown, NY, USA). The manipulation was performed following the manufacturer’s instructions.

### AFC Evaluation

This ultrasound examination was performed at the multi centers. All participating research institutes were modernized large comprehensive hospitals and had our regular supervision and verification. We formulated the unified standard for this examination in the beginning, and all ultrasound doctors were strictly trained and tested AFCs according to the same standard. AFC was regarded as the total number of visible, round or oval, intra-ovarian transonic follicles with a diameter ranging from 2 to 10 mm. Ultrasound examinations were performed by experienced fertility specialists in each of the participating research institutes. If one or both ovaries could not be spotted, the AFC was defined as not visible.

### Statistical Analysis

Shapiro-Wilk test was conducted to test the distribution types of continuous variables and found that they all conformed to skewed distributions. Therefore, they were presented as median and 90% prediction interval (5-95 percentiles). Variables, such as inhibin B and AMH, were also logarithmically transformed in the case of significant deviation from the normal distribution. The reference ranges were illustrated according to the value of 90th percentile, median and 10th percentile, as well as mean+2 SD and mean–2 SD, which has also been reported by a previous study (20). Inhibin B levels across different age groups (5-year intervals before 40 years old or 10-year intervals after 40 years old) in adult women were analyzed by the Kruskal-Wallis test, and the value for each group (each age interval) was compared with those of the previous group using the Mann-Whitney U test, which was also adopted in our previous study (16). Spearman correlation analyses were used in our study to calculate the relationships between inhibin B levels and age, BMI, as well as other ovarian reserve or function markers. As a novel method that has also been employed by other scholars (21), nomogram curves for the distribution of the inhibin B levels as a function of age were also calculated. All *P*-values were two-tailed, and values < 0.05 were considered statistically significant. Receiver operating characteristic (ROC) curves were plotted to evaluate the value of inhibin B and FSH in predicting AFC <5-7. All statistical analyses were carried out using the IBM SPSS Statistics 13.0 statistical software package (SPSS Inc., Chicago, IL, USA).

## Results

### The Basal Clinical Characteristics

The median age of the establishment population was 32.3 years (range 5%-95%, 22.7–44.4 years), and 28.7 years (range 5%-95%, 23.9-34.3 years) for the validation population. AFC, serum hormones, including inhibin B, FSH, LH, PRL, progesterone, E2, T, and AMH were measurable in the majority of individuals (**Table 1**).

**Table 1:** Median and 90% prediction interval (5–95 percentiles) of serum hormones and AFC in different age groups among the establishment population (n=948).

		Age groups					
		<25y	25–30y	30–35y	35–40y	> 40y	All
**Number**		200	200	200	202	146	948
Age(y)		23.7	27.6	32.3	37.3	42.5	32.3
5–95 percentiles		21.1-24.9	25.3-29.7	30.3-34.6	35.2-39.8	40.2-48.7	22.7-44.4
BMI (kg/m^2^)		19.8	20.4	20.9	22.1	23.0	21.3
5–95 percentiles		16.4-25.4	17.0-27.0	17.7-27.5	18.1-27.4	19.5-27.8	17.5-27.3
Inhibin B (pg/mL)		82.6	79.8	88.0	74.5	55.1	78.1
5–95 percentiles		26.8-137.1	39.8-160.4	35.6-139.1	27.8-127.1	2.6-135.5	12.1-137.4
_log_inhibin B (pg/mL)		4.4	4.4	4.5	4.3	4.0	4.4
5–95 percentiles		3.3-4.9	3.7-5.1	3.6-4.9	3.3-4.9	0.9-5.0	2.5-4.9
FSH (mIU/mL)		6.1	6.8	6.9	7.1	7.9	7.0
5–95 percentiles		3.8-9.5	4.3-10.5	4.6-10.0	4.8-11.2	4.6-26.4	4.4-12.5
LH (mIU/mL)		4.6	4.4	3.9	3.8	4.5	4.2
5–95 percentiles		2.1-10.4	2.2-9.9	1.8-8.6	1.7-8.9	1.7-12.1	1.9-10.0
Estradiol (pg/ml)		39.6	40.3	43.0	42.4	40.6	41.2
5–95 percentiles		21.5-75.3	16.9-91.7	15.8-89.0	14.0-91.0	12.3-203.0	16.6-96.1
AMH (ng/ml)		6.2	5.6	4.2	3.2	1.0	3.7
5–95 percentiles		2.1-12.7	1.6-13.3	1.4-11.6	0.6-9.5	0.1-5.5	0.1-11.7
_log_AMH (ng/ml)		0.8	0.7	0.6	0.5	0.01	0.6
5–95 percentiles		0.3-1.1	0.2-1.1	0.2-1.1	-0.2-1.0	-1.1-0.7	-1.1-1.1
PRL (ng/ml)		13.4	14.0	13.0	11.1	10.6	12.2
5–95 percentiles		7.1-39.7	7.3-29.9	6.3-32.1	5.7-26.6	5.3-28.4	6.2-30.2
PRG (ng/ml)		0.6	0.6	0.6	0.5	0.5	0.6
5–95 percentiles		0.1-1.3	0-1.5	0-1.2	0.1-1.0	0.2-1.3	0.1-1.2
T (ng/dl)		32.5	30.3	30.0	25.0	18.0	26.6
5–95 percentiles		9.9-61.1	9.0-60.9	6.5-58.7	2.5-46.2	0-41.7	2.5-55.9
AFCs		14	13	11	10	4	11
5–95 percentiles		8-24	5-22	5-20	2-18	0-14	2-21

BMI, body mass index; FSH, follicle-stimulating hormone; LH, luteinizing hormone; AMH, anti-Mullerian hormone; PRL, prolactin; PRG, progesterone; T, testosterone; AFC, antral follicle counts; y, year.

### The Variation Trend of Inhibin B in Healthy Women of All Ages

**Figure 2:**
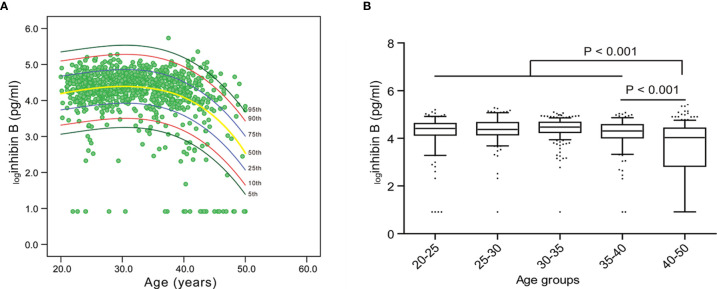
Nomogram for inhibin B and the variation trend of inhibin B with age. **(A)** Inhibin B nomogram based on the cubic regression model. Each line represents the value of confidential interval (CI). A rapid decrease of inhibin B value was observed in women after 40 years old. **(B)** Median value of inhibin B in each age group.

It can be deduced that the level of inhibin B rapidly decreases after the age of 40, based on the nomogram of the cubic regression model (**Table 1**, **Figure 2A**). We chose this model because it had the least sum of squared residuals compared with the linear regression model and the quadratic regression model. According to both the highest R^2^ and the convenience of interpretation, the cubic model was observed to be the best of these regression models and was chosen as the most appropriate one to illustrate the relationship between inhibin B and age. The confidence interval (CI) for 5%, 10%, 25%, 50%, 75%, 90% and 95% was also depicted in this nomogram.

The reference range of inhibin B for each age group was expressed as mean and median (**Table 1**, **Supplementary Tables 1, 2**). For women 20-25, 25-30, 30-35, 35-40, and ≥ 40 years old, the median value of inhibin B was 82.6 pg/ml, 79.8 pg/ml, 88.0 pg/ml, 74.5 pg/ml, and 55.1 pg/ml, respectively. The level of _log_inhibin B of 40-50 years old women was significantly lower than 35-40 years old women (mean 3.6 vs 4.3, median 4.03 *vs* 4.31, P<0.001). Furthermore, the value between the age group of 20-40 years old and 40-50 years old was also significantly different (mean 4.3 *vs* 3.6, median 4.41 *vs* 4.03, P<0.001) (**Figure 2B**).

### Relationship Between Inhibin B and Hormones Secreted by the Pituitary Gland

**Figure 3:**
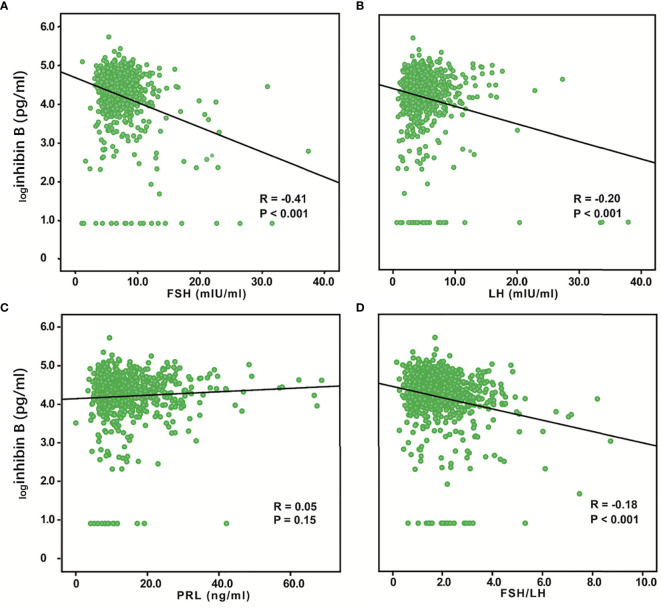
Relationship between serum inhibin B levels and FSH, LH, PRL, and FSH/LH. _log_Inhibin B was significantly negatively correlated with FSH (R = -0.41, P < 0.001) **(A)** and LH (R = -0.20, P < 0.001) **(B)**. No correlation was found between logInhibin B and PRL (R = 0.05, P =0.15) **(C)**. _log_Inhibin B was significantly negatively correlated with FSH/LH (R = -0.18, P < 0.001) **(D)**.

We evaluated the relationship between inhibin B and FSH, LH, and PRL. Inhibin B showed a significant negative correlation to FSH (R = -0.41, P < 0.001) and LH (R = -0.20, P < 0.001), while no correlation was found between Inhibin B and PRL (**Figures 3A, B, D**). We also analyzed the correlation between these hormones and age and found a significant positive correlation between FSH (R=0.30, P< 0.001) and age, while PRL (R=-0.17, P< 0.001) was negatively correlated with age, and no correlation was found between LH and age (**Supplementary Figure 1**). The previous study has found that FSH/LH ratio can forecast poor and excessive ovarian response in IVF-ICSI (22), so we also analyzed this variable, and found that FSH/LH was significantly negatively correlated with inhibin B (R=-0.18, P < 0.001), and was positively correlated with age (R=0.27, P< 0.001) (**Figure 3C**, **Supplementary Figure 1**).

### Relationship Between Inhibin B and AFC, Hormones Secreted by the Ovary

**Figure 4:**
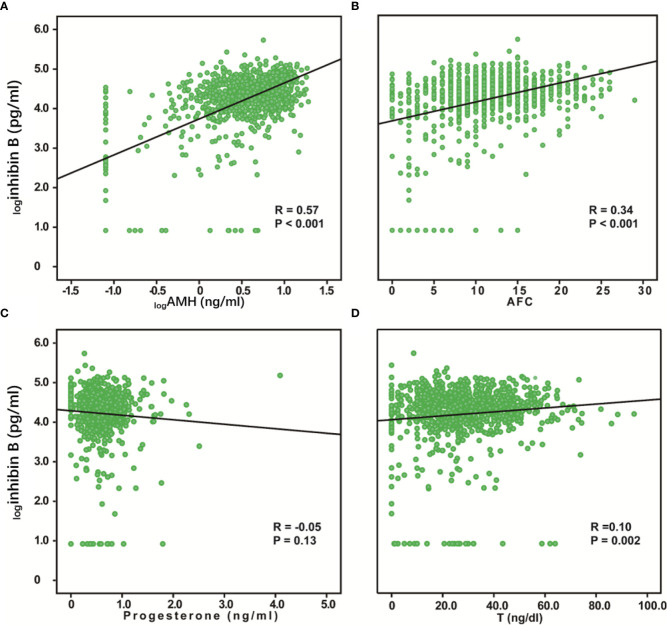
Relationship between serum inhibin B levels and AMH, AFC, Progesterone, and T. _log_Inhibin B was significantly positively correlated with _log_AMH (R = 0.57, P < 0.001) **(A)**, AFC (R =0.34, P < 0.001) **(B)**, and T (R = 0.10, P =0.002) **(D)**. No correlation was found between _log_Inhibin B and progesterone (R = -0.05, P =0.13) **(C)**.

We evaluated the relationship between inhibin B and AMH, AFC, progesterone, and T. AMH and AFC are classic markers for ovarian reserve, while progesterone and T are hormones secreted by ovaries, which can also reflect the ovarian function. Inhibin B was positively correlated with _log_AMH (R = 0.57, P < 0.001), AFC (R = 0.34, P < 0.001) and, T (R = 0.10, P = 0.002), while the association between inhibin B and progesterone was not significant (**Figure 4**). We also analyzed the relationship between these variables and age and found that _log_AMH (R=-0.54, P< 0.001), AFC (R=-0.52, P< 0.001), progesterone (R=-0.09, P=0.01) and T (R=-0.31, P< 0.001) were all significantly negatively correlated with age (**Supplementary Figure 1**).

### Inhibin B Showed Greater Potency in Predicting AFC﹤5-7 Compared to FSH

**Figure 5:**
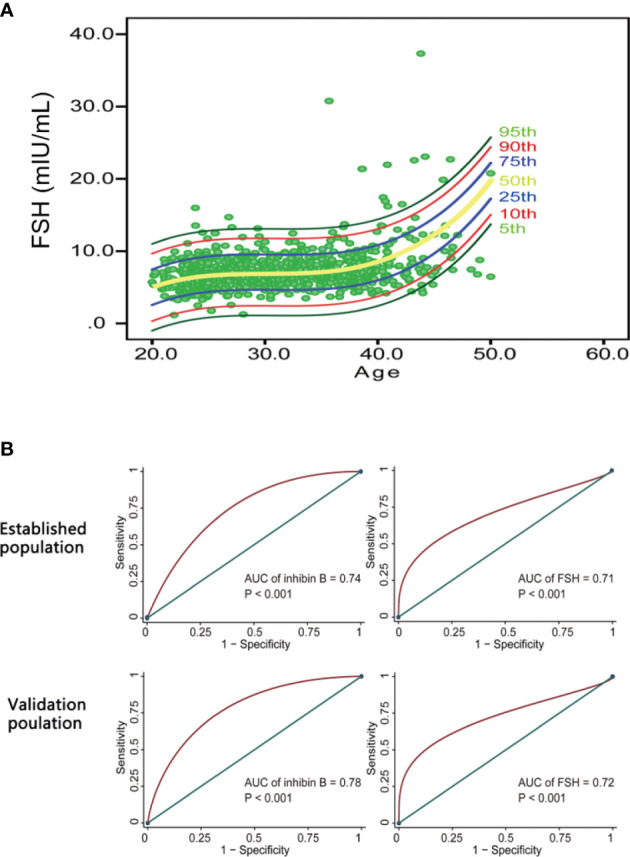
The potency of inhibin B and FSH in predicting AFC<5-7. ** (A)** FSH nomogram based on cubic regression model. Each line represents the value of confidential interval (CI). Rapid increase of FSH value was observed in women after 40 years old. **(B)** ROC curves evaluating the diagnostic value of inhibin B and FSH in predicting AFC < 5-7. Inhibin B (AUC = 0.74, P < 0.001 for the establishment population; AUC = 0.78, P < 0.001 for the validation population) had a slightly higher value than FSH (AUC = 0.71, P < 0.001 for the establishment population; AUC = 0.72, P < 0.001 for the validation population) in diagnosing AFC<5-7.

Because FSH showed a significant negative correlation with inhibin B, a nomogram was also built based on the cubic regression model. It can also be deduced that FSH rapidly increases after approximately 40 years of age (**Figure 5A**). The cubic model was chosen as the most appropriate model to illustrate the relationship between FSH and age because it had both the highest R^2^ and the least sum of squared residuals and is convenient to interpret compared with the linear regression model and quadratic regression model. The confidence interval (CI) for 5%, 10%, 25%, 50%, 75%, 90% and 95% are also depicted in this nomogram. It can be speculated that there are more women over 40 years old with low levels of inhibin B and high levels of FSH.

According to the ‘Bologna’ criteria, AFC less than 5-7 follicles is one of the diagnosis criteria for poor ovarian response (23). Therefore, we indirectly compared the ability of inhibin B and FSH to predict ovarian response by comparing their predictive ability to AFC < 5-7. The data in the establishment group and validation group were employed to calculate the area under the ROC curve (AUC). Inhibin B (AUC = 0.74, P < 0.001 for the establishment population; AUC = 0.78, P < 0.001 for the validation population) had a slightly higher value than FSH (AUC = 0.71, P < 0.001 for the establishment population; AUC = 0.72, P < 0.001 for the validation population) in predicting AFC < 5-7 (**Figure 5B**).

## Discussion

The variation trend of inhibin B was detected and a normal reference range was successfully established in healthy reproductive age Chinese women, which was also validated in a group of women less than 40 years old. We found that inhibin B levels were detectable in 95% of the individuals, and the levels were nearly the same among women 20-35 years old, but significantly decreased in adult women over 40 years old. This finding is consistent with the previous finding that women’s ovarian reserve and fertility decreased at a drastic speed after approximately 37.5 years (24).

Although the reference range of inhibin B remains unclear, the data is still rare, inhibin B has already been widely studied for its important role in the regulation of the hypothalamus-pituitary-gonadal axis through suppressive effects on activin-mediated FSH expression and release, and the direct effects on ovarian folliculogenesis, steroidogenesis and menstrual cycle, which can affect AFC results (25). FSH and AFC are traditional ovarian reserve indicators, therefore, inhibin B may have a potential role in reflecting ovarian reserve too. Our results on the normal reference range of inhibin B may contribute to a more accurate evaluation of ovarian reserve. Besides, FSH is an indirect marker of ovarian reserve and influenced by hypothalamic function, ovarian factors, and steroid hormones. In contrast, the inhibin B concentration would be a more direct marker of the ovarian reserve because it is produced by small ovarian follicles and is therefore direct measures of the follicular pool (26, 27).

In addition to finding that the inhibin B levels of reproductive age women decreased with age, our study also revealed that serum inhibin B levels were significantly positively correlated with AMH and AFC, and negatively correlated with age, FSH, LH, and FSH/LH. These results were in accord with the previous studies (4, 18, 28, 29) (**Supplementary Tables 3, 4**). Although some research conclusions were still controversial, most studies on women with impaired ovarian function, including POF or POI patients (5, 6, 30–32), diminished ovarian reserve (DOR) patients (33, 34), and cancer survivors (7, 8), also found a dramatic decrease in inhibin B, most of them were below the detection limit (**Supplementary Table 5**). These pieces of evidence further indicate that inhibin B has a certain potential in evaluating ovarian reserve.

However, inhibin B is currently not a reliable measure of ovarian reserve in clinical practice. The main reasons may be as follows. Firstly, Inhibin B concentrations fluctuate with the menstrual cycle and ART cycle (35, 36) (**Supplementary Table 3**). The previous study has found that the concentration of inhibin B rose rapidly in the early follicular phase to a peak on the day after the intercycle FSH rise, then fell progressively during the remainder of the follicular phase, two days after the midcycle LH peak, there was a short-lived peak in the inhibin B concentration, which then fell to a low concentration for the remainder of the luteal phase (35). Secondly, due to different populations and detection kits in different studies, the absolute values of inhibin B concentrations were also different. Thus, clinicians may find it difficult to generalize inhibin B cut-off values in the medical literature to clinical practices unless they are using the very same assay and reference preparation. Further efforts are needed to standardize the detection technology of inhibin B. Thirdly, limited by small sizes, heterogeneity among study design, analyses and outcomes, and the lack of validated outcome measures, the ability of inhibin B to assess ovarian reserve is still controversial. More prospective studies with larger sample sizes are needed to provide more reliable evidence.

Despite recent striking advances in ART, poor ovarian reserve diagnosis and treatment is still considered challenging. The core of the pathophysiology of poor ovarian response is the limited number of follicles responding to FSH (37). Because our subjects had not undergone ART, we were not able to directly evaluate the response of their ovaries to controlled superovulation. Therefore, we changed our thinking and indirectly compared the ability of inhibin B and FSH to predict ovarian response by comparing their predictive ability to AFC < 5-7, which is one of the diagnostic criteria of poor ovarian response (23). In the end, we found that inhibin B had a slight advantage over FSH in predicting AFC < 5-7, suggesting that inhibin B may also have a role in evaluating ovarian response in women. Some previous studies also supported the value of inhibin B in predicting ovarian response and ART outcomes (11, 14, 15, 38–47), but the conclusions were still controversial (12, 13, 48, 49) (**Supplementary Table 6**). Moreover, a recent study on older reproductive age women found no association between inhibin B and reduced fertility (50). Collectively, more studies are needed, especially prospective studies with large sample sizes, to further clarify the relationship between inhibin B and natural fertility and ART outcomes.

This study had several strengths. The first one was the study design, which was a multi-center study with large sample size. We recruited a group of healthy reproductive age Chinese women through advertisements to establish an ovarian reserve database that included clinical and biological factors. Volunteers came from a nationwide region including six universities and eight medical institutes. Secondly, by analyzing and comparing the outcome data of 1553 health women in different age groups, we successfully established a reference range for inhibin B and established different levels of correlations among different ovarian reserve markers, explore the potency of inhibin B in evaluating ovarian reserve and ovarian response. Thirdly, updated ELISA kits were applied in our study. The new kits had new standards for measurement and had been renewed with higher accuracy and sensitivity. Therefore, samples that were undetectable in the past can now be detected with the new kits. However, the limitations of the current study deserve careful consideration. Firstly, our study only included healthy reproductive age women, while prepubescent, adolescent girls, and unhealthy adult women were not included. Secondly, we only tested the levels of serum inhibin B in the follicular phase, and there was no information for the variability of inhibin B within a menstrual cycle. Thirdly, there was a lack of data on fertility and ART outcomes, so it was impossible to directly assess the predictive value of inhibin B on fertility and ART outcomes.

## Conclusions

A total of 2,524 healthy reproductive age women from six universities and eight medical institutes participated in this recruitment. The reference range for serum inhibin B was established and validated among healthy adult women. For healthy reproductive age women, the decline of serum inhibin B can reflect decreased ovarian reserve effectively, having a good consistency with AMH and AFC. More importantly, inhibin B had a slight advantage in predicting AFC < 5-7 compared with FSH, which suggested the potential of inhibin B in predicting ovarian response. These results will be helpful to the clinical application of inhibin B in the evaluation of female ovarian reserve and the assessment of their reproductive capacity.

## Data Availability Statement

The data that support the findings of this study are available from the corresponding authors upon reasonable request.

## Ethics Statement

The studies involving human participants were reviewed and approved by the Ethical Committee of Tongji Hospital, Tongji Medical College, Huazhong University of Science and Technology. The patients/participants provided their written informed consent to participate in this study. The patients/participants provided their written informed consent for potentially identifiable human images or data presented in this study.

## Author Contributions

JW and JZ contributed to conceptualization, data curation, methodology, supervision, and writing the original draft. XD, HZ, TD, CZ, WM, YZ, WQ, YL, ZL, SD, YJ and AL contributed to healthy population recruitment and data collection. KH contributed to formal analysis, methodology, conceptualization, data curation, and writing the original draft. SW contributed to conceptualization, data curation, methodology, and supervision. All authors contributed to the article and approved the submitted version.

## Funding

This research was supported by the grant from the International S&T Cooperation Program of China (No.2013DFA31400), and grants from the National Natural Science Foundation of China (No.81673194, No.81873824, No.81300453).

## Conflict of Interest

The authors declare that the research was conducted in the absence of any commercial or financial relationships that could be construed as a potential conflict of interest.

## Supplementary Material

Supplementary Figure 1 The levels of hormones and BMI changed with age. _log_AMH (R = -0.54, P < 0.001) **(A)**, AFC (R =-0.52, P < 0.001) **(C)**, progesterone (R = -0.09, P =0.01) **(G)**, PRL (R = -0.17, P < 0.001) **(H)**, and T (R = -0.31, P < 0.001) **(I)** were significantly negatively correlated with age. FSH (R = 0.30, P < 0.001) **(B)**, BMI (R = 0.32, P < 0.001) **(D)**, and FSH/LH (R = 0.27, P < 0.001) **(E)** were significantly positively correlated with age. No correlation was found between LH (R = 0.03, P =0.41) **(F)** and age.
