# Supplementary figures and images for: Can Inhibin B Reflect Ovarian Reserve of Healthy Reproductive Age Women Effectively?
Source: Front Endocrinol (Lausanne). 2021 Apr 14;12:626534. doi: 10.3389/fendo.2021.626534 (PMC8081350; doi:10.3389/fendo.2021.626534)

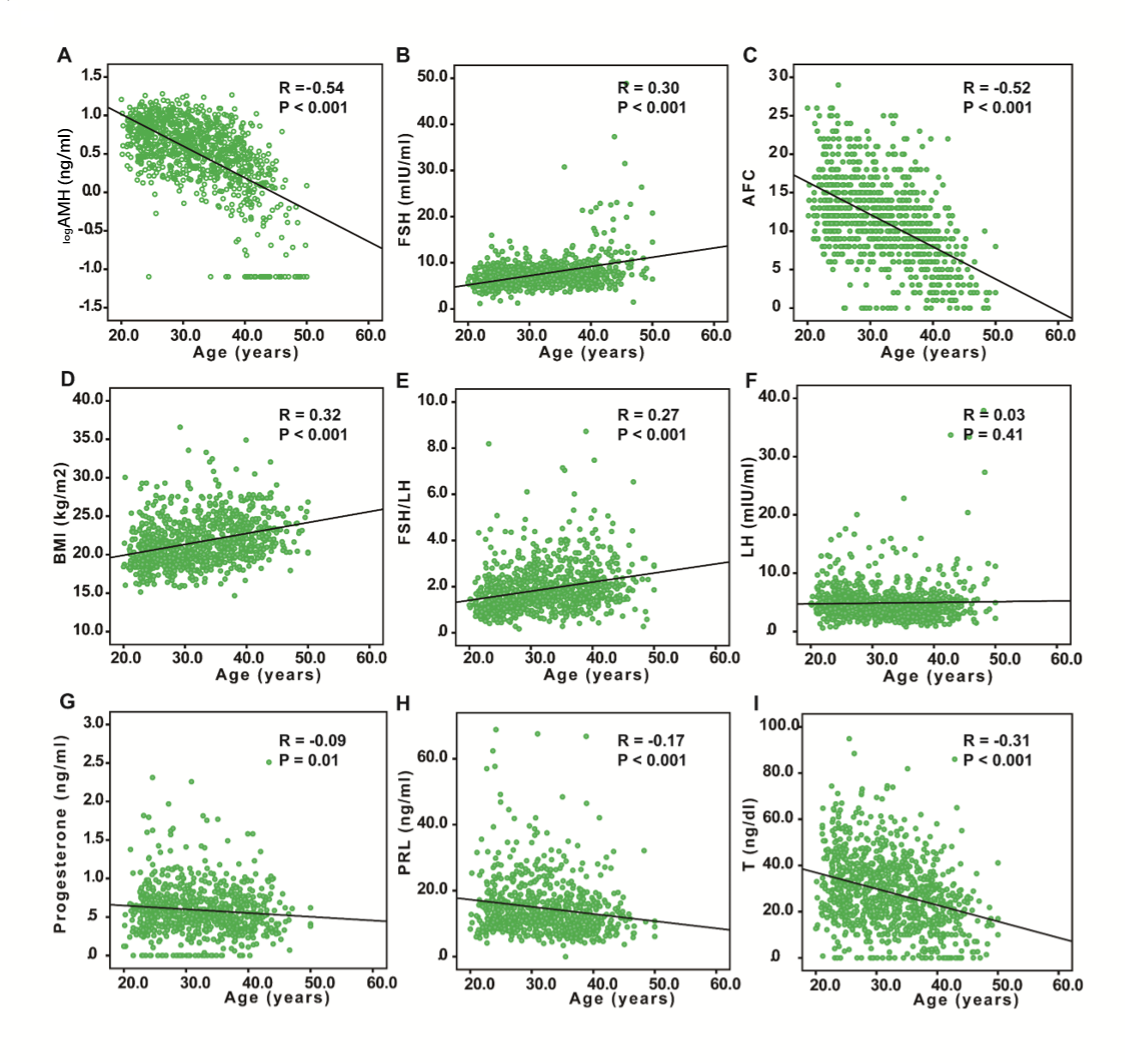

Supplement: Supplementary Figure 1 — The levels of hormones and BMI changed with age. logAMH (R = -0.54, P < 0.001) (A), AFC (R =-0.52, P < 0.001) (C), progesterone (R = -0.09, P =0.01) (G), PRL (R = -0.17, P < 0.001) (H), and T (R = -0.31, P < 0.001) (I) were significantly negatively correlated with age. FSH (R = 0.30, P < 0.001) (B), BMI (R = 0.32, P < 0.001) (D), and FSH/LH (R = 0.27, P < 0.001) (E) were significantly positively correlated with age. No correlation was found between LH (R = 0.03, P =0.41) (F) and age. [file Image_1.tif]
