# Supplementary material for: Can Inhibin B Reflect Ovarian Reserve of Healthy Reproductive Age Women Effectively?
Source: Front Endocrinol (Lausanne). 2021 Apr 14;12:626534. doi: 10.3389/fendo.2021.626534 (PMC8081350; doi:10.3389/fendo.2021.626534)
Supplement: Supplementary file 2 [file DataSheet_1.docx]

Supplementary Table 1. Mean value of _log_inhibin B (pg/mL) according to the age groups in the establishment (n=948) and validation (n=605) population.

| Age groups | Mean (±2 SD) | Mean (±2 SD) | |
| --- | --- | --- | --- |
|  |  | Establishment | Validation |
| 20-25y | 4.3±2*0.6 | 4.3±2*0.6 | 4.4±2*0.5 |
| 25-30y | 4.4±2*0.5 |  |  |
| 30-35y | 4.4±2*0.5 |  |  |
| 35-40y | 4.3±2*0.6 |  |  |
| ≥40y | 3.6±2*1.3 | 3.6±2*1.3 | NA |

SD, standard deviation; y, year.

Supplementary Table 2. Median value of _log_inhibin B (pg/mL) according to the age groups in the establishment (n=948) and validation (n=605) population.

| Age groups | Median (10-90 pc) | Median (10-90 pc) | |
| --- | --- | --- | --- |
|  |  | Establishment | Validation |
| 20-25y | 4.41 (3.81-4.80) | 4.41 (3.83-4.89) | 4.44 (3.87-4.83) |
| 25-30y | 4.38 (3.86-4.91) |  |  |
| 30-35y | 4.48 (3.94-4.86) |  |  |
| 35-40y | 4.31 (3.77-4.77) |  |  |
| ≥40y | 4.03 (0.92-4.74) | 4.03 (0.92-4.74) | NA |

y, year.
