# Supplementary material for: Can Inhibin B Reflect Ovarian Reserve of Healthy Reproductive Age Women Effectively?
Source: Front Endocrinol (Lausanne). 2021 Apr 14;12:626534. doi: 10.3389/fendo.2021.626534 (PMC8081350; doi:10.3389/fendo.2021.626534)
Supplement: Supplementary file 3 [file DataSheet_2.doc]

Table S3. Changes in inhibin B levels with age, menstrual cycle, or ovarian aging.

**References**

1. Sowers MR, Eyvazzadeh AD, McConnell D, Yosef M, Jannausch ML, Zhang D, et al. Anti-mullerian hormone and inhibin B in the definition of ovarian aging and the menopause transition. *J Clin Endocrinol Metab*. 2008;93(9):3478-3483. doi: 10.1210/jc.2008-0567.
2. Chellakooty M, Schmidt IM, Haavisto AM, Boisen KA, Damgaard IN, Mau C, et al. Inhibin A, inhibin B, follicle-stimulating hormone, luteinizing hormone, estradiol, and sex hormone-binding globulin levels in 473 healthy infant girls. *J Clin Endocrinol Metab*. 2003;88(8):3515-3520. doi: 10.1210/jc.2002-021468.
3. Sehested A, Juul AA, Andersson AM, Petersen JH, Jensen TK, Müller J, et al. Serum inhibin A and inhibin B in healthy prepubertal, pubertal, and adolescent girls and adult women: relation to age, stage of puberty, menstrual cycle, follicle-stimulating hormone, luteinizing hormone, and estradiol levels. *J Clin Endocrinol Metab*. 2000;85(4):1634-1640. doi: 10.1210/jcem.85.4.6512.
4. Welt CK, McNicholl DJ, Taylor AE, Hall JE. Female reproductive aging is marked by decreased secretion of dimeric inhibin. *J Clin Endocrinol Metab*. 1999;84(1):105-111. doi: 10.1210/jcem.84.1.5381.
5. Danforth DR, Arbogast LK, Mroueh J, Kim MH, Kennard EA, Seifer DB, et al. Dimeric inhibin: a direct marker of ovarian aging. *Fertil Steril*. 1988;70(1):119-123. doi: 10.1016/s0015-0282(98)00127-7.
6. Groome NP, Illingworth PJ, O'Brien M, Pai R, Rodger FE, Mather JP, et al. Measurement of dimeric inhibin B throughout the human menstrual cycle. *J Clin Endocrinol Metab*. 1996;81(4):1401-1405. doi: 10.1210/jcem.81.4.8636341
7. Klein NA, Battaglia DE, Miller PB, Branigan EF, Giudice LC, Soules MR. Decreased inhibin B secretion is associated with the monotropic FSH rise in older, ovulatory women: a study of serum and follicular fluid levels of dimeric inhibin A and B in spontaneous menstrual cycles. *J Clin Endocrinol Metab*. 1996;81(7):2742-2745. doi: 10.1210/jcem.81.7.8675606.
8. Groome NP, Illingworth PJ, O'Brien M, Cooke I, Ganesan TS, Baird DT, et al. Detection of dimeric inhibin throughout the human menstrual cycle by two-site enzyme immunoassay. *Clin Endocrinol (Oxf)*. 1994;40(6):717-723. doi: 10.1111/j.1365-2265.1994.tb02504.x.

Table S4. The value of inhibin B in evaluating ovarian reserve and fertility.

**References**

1. Steiner AZ, Pritchard D, Stanczyk FZ, Kesner JS, Meadows JW, Herring AH, et al. Association Between Biomarkers of Ovarian Reserve and Infertility Among Older Women of Reproductive Age. *JAMA*. 2017;318(14):1367-1376. doi: 10.1001/jama.2017.14588.
2. Fanchin R, Schonauer LM, Righini C, Guibourdenche J, Frydman R, Taieb J. Serum anti-Mullerian hormone is more strongly related to ovarian follicular status than serum inhibin B, estradiol, FSH and LH on day 3. *Hum Reprod*. 2003;18(2):323-327. doi: 10.1093/humrep/deg042.
3. Tinkanen H, Bläuer M, Laippala P, Tuohimaa P, Kujansuu E. Correlation between serum inhibin B and other indicators of the ovarian function. *Eur J Obstet Gynecol Reprod Biol*. 2001;94(1):109-113. doi: 10.1016/s0301-2115(00)00319-5.
4. Luborsky J, Llanes B, Roussev R, Coulam C. Ovarian antibodies, FSH and inhibin B: independent markers associated with unexplained infertility. *Hum Reprod*. 2000;15(5):1046-1051. doi: 10.1093/humrep/15.5.1046.
5. Corson SL, Gutmann J, Batzer FR, Wallace H, Klein N, Soules MR. Inhibin-B as a test of ovarian reserve for infertile women. *Hum Reprod*. 1999;14(11):2818-2821. doi: 10.1093/humrep/14.11.2818.

Table S5. Changes in inhibin B levels in women with impaired ovarian function.

POI, Premature ovarian insufficiency; bPOI, biochemical POI; CCS, Childhood cancer survivors; DOR, Diminished ovarian reserve; LPD, Luteal phase deficiency; SP, Spontaneous puberty; TS, Turner syndrome.

**References**

1. Zhu C, Luo W, Li Z, Zhang X, Hu J, Zhao S, et al. New theca-cell marker insulin-like factor 3 is associated with premature ovarian insufficiency. *Fertil Steril*. 2020. doi: 10.1016/j.fertnstert.2020.08.005.
2. Ruszala A, Wojcik M, Starzyk J. Evaluation of the usefulness of antymullerian hormone and inhibin B as markers of ovarian activity in patients with Turner syndrome - preliminary results. *Pediatr Endocrinol Diabetes Metab*. 2020;26(2):84-88. doi: 10.5114/pedm.2020.95622.
3. Pfister A, Crawford NM, Steiner AZ. Association between diminished ovarian reserve and luteal phase deficiency. *Fertil Steril*. 2019;112(2):378-386. doi: 10.1016/j.fertnstert.2019.03.032.
4. Nystrom A, Morse H, Nordlof H, Wiebe K, Artman M, Ora I, et al. Anti-mullerian hormone compared with other ovarian markers after childhood cancer treatment. *Acta Oncol*. 2019;58(2):218-224. doi: 10.1080/0284186X.2018.1529423.
5. van den Berg MH, Overbeek A, Lambalk CB, Kaspers GJL, Bresters D, van den Heuvel-Eibrink MM, et al. Long-term effects of childhood cancer treatment on hormonal and ultrasound markers of ovarian reserve. *Hum Reprod*. 2018;33(8):1474-1488. doi: 10.1093/humrep/dey229.
6. Bidet M, Bachelot A, Bissauge E, Golmard JL, Gricourt S, Dulon J, et al. Resumption of ovarian function and pregnancies in 358 patients with premature ovarian failure. *J Clin Endocrinol Metab*. 2011;96(12):3864-3872. doi: 10.1210/jc.2011-1038.
7. Li HW, Anderson RA, Yeung WS, Ho PC, Ng EH. Evaluation of serum antimullerian hormone and inhibin B concentrations in the differential diagnosis of secondary oligoamenorrhea. *Fertil Steril*. 2011;96(3):774-779. doi: 10.1016/j.fertnstert.2011.06.016.
8. Su HI, Sammel MD, Green J, Velders L, Stankiewicz C, Matro J, et al. Antimullerian hormone and inhibin B are hormone measures of ovarian function in late reproductive-aged breast cancer survivors. *Cancer*. 2010;116(3):592-599. doi: 10.1002/cncr.24746.
9. Knauff EA, Eijkemans MJ, Lambalk CB, ten Kate-Booij MJ, Hoek A, Beerendonk CC. et al. Anti-Mullerian hormone, inhibin B, and antral follicle count in young women with ovarian failure. *J Clin Endocrinol Metab*. 2009;94(3):786-792. doi: 10.1210/jc.2008-1818.
10. Tsigkou A, Marzotti S, Borges L, Brozzetti A, Reis F, Candeloro P, et al. High serum inhibin concentration discriminates autoimmune oophoritis from other forms of primary ovarian insufficiency. *J Clin Endocrinol Metab*. 2008;93(4):1263-1269. doi: 10.1210/jc.2007-1675.
11. Welt CK, Hall JE, Adams JM, Taylor AE. Relationship of estradiol and inhibin to the follicle-stimulating hormone variability in hypergonadotropic hypogonadism or premature ovarian failure. *J Clin Endocrinol Metab.* 2005;90(2):826-830. doi: 10.1210/jc.2004-1319.
12. Munz W, Hammadeh ME, Seufert R, Schaffrath M, Schmidt W, Pollow K. Serum inhibin A, inhibin B, pro-alphaC, and activin A levels in women with idiopathic premature ovarian failure. *Fertil Steril*. 2004;82(3):760-762. doi: 10.1016/j.fertnstert.2004.05.065.
13. Erdem M, Erdem A, Gursoy R, Biberoglu K. Comparison of basal and clomiphene citrate induced FSH and inhibin B, ovarian volume and antral follicle counts as ovarian reserve tests and predictors of poor ovarian response in IVF. *J Assist Reprod Genet.* 2004;21(2):37-45. doi: 10.1023/b:jarg.0000025936.73125.b4.
14. Bath LE, Wallace WH, Shaw MP, Fitzpatrick C, Anderson RA. Depletion of ovarian reserve in young women after treatment for cancer in childhood: detection by anti-Mullerian hormone, inhibin B and ovarian ultrasound. *Hum Reprod*. 2003;18(11):2368-2374. doi: 10.1093/humrep/deg473.
15. Seifer DB, Scott RT Jr, Bergh PA, Abrogast LK, Friedman CI, Mack CK, et al. Women with declining ovarian reserve may demonstrate a decrease in day 3 serum inhibin B before a rise in day 3 follicle-stimulating hormone. *Fertil Steril*. 1999;72(1):63-65. doi: 10.1016/s0015-0282(99)00193-4.

Table S6. The value of inhibin B in predicting ovarian response and ART outcomes.

ART, Assisted reproductive technology; COH, Controlled ovarian hyperstimulation; DOR, Diminished ovarian reserve; GnRH, Gonadotropin releasing hormone; ICSI, Intracytoplasmic sperm injection; IVF, In vitro fertilization; OHSS, ovarian hyperstimulation syndrome; PCOS, Polycystic ovary syndrome.

**References**

1. Lawrenz B, Depret Bixio L, Coughlan C, Andersen CY, Melado L, Kalra B, et al. Inhibin A-A Promising Predictive Parameter for Determination of Final Oocyte Maturation in Ovarian Stimulation for IVF/ICSI. *Front Endocrinol (Lausanne)*. 2020;11:307. doi: 10.3389/fendo.2020.00307.
2. Li Y, Nie M, Liu Y, Zhang W, Yang X. The dynamic changes of anti-Mullerian hormone and inhibin B during controlled ovarian hyperstimulation in decreased ovarian reserve women and the effect on clinical outcome. *Gynecol Endocrinol*. 2015;31(6):450-453. doi: 10.3109/09513590.2014.998187.
3. Ocal P, Sahmay S, Cetin M, Irez T, Guralp O, Cepni I. Serum anti-Mullerian hormone and antral follicle count as predictive markers of OHSS in ART cycles. *J Assist Reprod Genet.* 2011;28(12):1197-1203. doi: 10.1007/s10815-011-9627-4.
4. Lie Fong S, Schipper I, de Jong FH, Themmen AP, Visser JA, Laven JS. Serum anti-Mullerian hormone and inhibin B concentrations are not useful predictors of ovarian response during ovulation induction treatment with recombinant follicle-stimulating hormone in women with polycystic ovary syndrome. *Fertil Steril.* 2011;96(2):459-463. doi: 10.1016/j.fertnstert.2011.05.084.
5. Penarrubia J, Peralta S, Fabregues F, Carmona F, Casamitjana R, Balasch J. Day-5 inhibin B serum concentrations and antral follicle count as predictors of ovarian response and live birth in assisted reproduction cycles stimulated with gonadotropin after pituitary suppression. *Fertil Steril.* 2010;94(7):2590-2595. doi: 10.1016/j.fertnstert.2010.03.009.
6. Jayaprakasan K, Campbell B, Hopkisson J, Johnson I, Raine-Fenning N. A prospective, comparative analysis of anti-Mullerian hormone, inhibin-B, and three-dimensional ultrasound determinants of ovarian reserve in the prediction of poor response to controlled ovarian stimulation. *Fertil Steril.* 2010;93(3):855-864. doi: 10.1016/j.fertnstert.2008.10.042.
7. Miao MF, Huang HF. Dynamic measurements of serum inhibin B and estradiol: a predictive evaluation of ovarian response to gonadotrophin stimulation in the early stage of IVF treatment. *J Zhejiang Univ Sci B.* 2009;10(1):35-45. doi: 10.1631/jzus.B0820029.
8. Decanter C, Pigny P, Lefebvre C, Thomas P, Leroy M, Dewailly D. Serum inhibin B during controlled ovarian hyperstimulation: an additional criterion for deciding whether to proceed with egg retrieval. *Fertil Steril.* 2009;91(6):2419-2425. doi: 10.1016/j.fertnstert.2008.03.037.
9. Riggs RM, Duran EH, Baker MW, Kimble TD, Hobeika E, Yin L, et al. Assessment of ovarian reserve with anti-Mullerian hormone: a comparison of the predictive value of anti-Mullerian hormone, follicle-stimulating hormone, inhibin B, and age. *Am J Obstet Gynecol.* 2008;199(2):202 e201-208. doi: 10.1016/j.ajog.2008.05.004.
10. Muttukrishna S, McGarrigle H, Wakim R, Khadum I, Ranieri DM, Serhal P. Antral follicle count, anti-mullerian hormone and inhibin B: predictors of ovarian response in assisted reproductive technology? *BJOG.* 2005;112(10):1384-1390. doi: 10.1111/j.1471-0528.2005.00670.x.
11. Eldar-Geva T, Ben-Chetrit A, Spitz IM, Rabinowitz R, Markowitz E, Mimoni T, et al. Dynamic assays of inhibin B, anti-Mullerian hormone and estradiol following FSH stimulation and ovarian ultrasonography as predictors of IVF outcome. *Hum Reprod.* 2005;20(11):3178-3183. doi: 10.1093/humrep/dei203.
12. Urbancsek J, Hauzman E, Klinga K, Rabe T, Papp Z, Strowitzki T. Use of serum inhibin B levels at the start of ovarian stimulation and at oocyte pickup in the prediction of assisted reproduction treatment outcome. *Fertil Steril.* 2005;83(2):341-348. doi: 10.1016/j.fertnstert.2004.06.065.
13. Muttukrishna S, Suharjono H, McGarrigle H, Sathanandan M. Inhibin B and anti-Mullerian hormone: markers of ovarian response in IVF/ICSI patients? *BJOG.* 2004;111(11):1248-1253. doi: 10.1111/j.1471-0528.2004.00452.x.
14. Erdem M EA, Gursoy R, Biberoglu K. Comparison of basal and clomiphene citrate induced FSH and inhibin B, ovarian volume and antral follicle counts as ovarian reserve tests and predictors of poor ovarian response in IVF. *J Assist Reprod Genet.* 2004;21(2):37-45. doi: 10.1023/b:jarg.0000025936.73125.b4.
15. Kokcu A, Turhan E, Cetinkaya MB, Yanik F, Alper T, Malatyalioglu E. Inhibin B levels on cycle day 3 to predict the ovulatory response in women with PCOS undergoing ovulation induction via low dose step-up gonadotropin protocol. *Arch Gynecol Obstet.* 2004;270(4):255-259. doi: 10.1007/s00404-003-0557-y.
16. Fried G, Remaeus K, Harlin J, Krog E, Csemiczky G, Aanesen A, et al. Inhibin B predicts oocyte number and the ratio IGF-I/IGFBP-1 may indicate oocyte quality during ovarian hyperstimulation for in vitro fertilization. *J Assist Reprod Genet.* 2003;20(5):167-176. doi: 10.1023/a:1023656225053.
17. Fiçicioğlu C KT, Demirbaşoğlu S, Mulayim B. The role of inhibin B as a basal determinant of ovarian reserve. *Gynecol Endocrinol.* 2003;17(4):287-293.
18. Peñarrubia J, Balasch J, Fábregues F, Carmona F, Casamitjana R, Moreno V, et al. Day 5 inhibin B serum concentrations as predictors of assisted reproductive technology outcome in cycles stimulated with gonadotrophin-releasing hormone agonist-gonadotrophin treatment. *Hum Reprod.* 2000;15(7):1499-1504. doi: 10.1093/humrep/15.7.1499.
19. Hall JE, Welt CK, Cramer DW. Inhibin A and inhibin B reflect ovarian function in assisted reproduction but are less useful at predicting outcome. *Hum Reprod.* 1999;14(2):409-415. doi: 10.1093/humrep/14.2.409.
20. Seifer DB, Lambert-Messerlian G, Hogan JW, Gardiner AC, Blazar AS, Berk CA. Day 3 serum inhibin-B is predictive of assisted reproductive technologies outcome. *Fertil Steril.* 1997;67(1):110-114. doi: 10.1016/s0015-0282(97)81865-1.
